# Regulation of Root Nodule Symbiosis by Soybean *Rj* Genotypes and Rhizobial Effectors

**DOI:** 10.1264/jsme2.ME25027

**Published:** 2025-08-23

**Authors:** Shogo Fukunaga, Safirah Tasa Nerves Ratu, Shin Okazaki

**Affiliations:** 1 Graduate School of Agriculture, Tokyo University of Agriculture and Technology, Saiwaicho 3–5–8, Fuchu, Tokyo 183–8509, Japan; 2 Institute of Global Innovation Research, Tokyo University of Agriculture and Technology, Saiwaicho 3–5–8, Fuchu, Tokyo 183–8509, Japan

**Keywords:** *Rj* genotype, soybean, *Bradyrhizobium*, type III secretion system

## Abstract

Soybean (*Glycine max*) is one of the most important crops worldwide. Root nodule symbiosis between soybean and rhizobia has been extensively exami­ned due to its significance for agricultural productivity and environmental sustainability. Recent advances have enhanced our understanding of the soybean genotypes known as the *Rj*/*rj* genotypes, which play a critical role in regulating root nodule symbiosis. Furthermore, the function of rhizobium-secreted proteins, termed effectors, in eliciting specific responses in soybean *Rj*/*rj* genotypes has been elucidated. This review summarizes the involvement of soybean *Rj*/*rj* genotypes and their corresponding root nodule bacterial effectors in the regulation of nodule formation. We also discussed the potential for manipulating root nodule symbiosis by applying *Rj*/*rj* genotypes in soybean breeding programs, which may enhance nitrogen fixation efficiency and subsequently reduce the need for chemical fertilizers and greenhouse gas emissions from agricultural land.

Soybean (*Glycine max* [L.] Merr.), a leguminous crop, is one of the most essential agricultural products worldwide and is extensively utilized for food production, oil extraction, and animal feed (Rotundo *et al.*, 2024). Soybeans establish root nodules through a symbiotic relationship with rhizobia, a type of nitrogen-fixing bacteria, which facilitates the conversion of atmospheric nitrogen into bioavailable nitrogen sources (Zahran, 1999).

Extensive research has focused on soybean growth and yield, investigating both the plant and its symbiotic rhizobia, including the *Bradyrhizobium* and *Sinorhizobium* (*Ensifer*) groups (Caldwell, 1966; Vest, 1970; Vest and Caldwell, 1972; Devine and Kuykendall, 1994; Zhang *et al.*, 2021). Specific soybean cultivars and their mutant lines have exhibited either the suppression or enhancement of nodulation (Pracht *et al.*, 1993; Vuong *et al.*, 1996; Hayashi *et al.*, 2012) (Table 1). These symbiotic phenotypes are determined by *Rj* genotypes (dominant *Rj* and recessive *rj* alleles) and have been the focus of global research for more than 70 years (Williams and Lynch, 1954; Zhang *et al.*, 2021).

Ten *Rj* genotypes have been identified to date, with ongoing studies investigating the factors and mechanisms involved in the interactions between soybean and rhizobia (Table 1). This review aims to: (1) summarize existing research on *Rj* genotypes, (2) describe the rhizobial factors involved in these interactions, and (3) discuss the potential agricultural applications of symbiotic control mechanisms in soybean and rhizobia.

## Discovery of *Rj* genotypes in soybean

In early August 1947, the soybean breeding line L6-1743 (Lincoln [Lincoln×Richland]) cultivated in Urbana, Illinois, USA, displayed yellow leaves, in contrast to other green-leafed lines. Initially presumed to be the result of a chlo­rophyll-related mutation, subsequent investigations revealed nitrogen deficiency due to its nodulation-deficient characteristics. A genetic ana­lysis through backcrossing confirmed that this nodulation-deficient trait was associated with a recessive *rj*_1_ allele (Williams and Lynch, 1954). The L6-1743 *rj*_1_ line, exhibiting symbiotic incompatibility with a wide range of rhizobial strains, became the first reported *Rj* genotype.

Following the identification of *rj*_1_ in 1954, a novel nodulation-restriction trait was reported in the soybean cultivar Hardee in association with *Bradyrhizobium diazoefficiens* USDA122 (Caldwell *et al.*, 1966). A genetic ana­lysis through backcrossing revealed that this nodulation-restriction trait was a dominant allele (Caldwell, 1966). This nodulation-restriction trait with Hardee only occurred in specific rhizobial strains and exhibited different characteristics to *rj*_1_; therefore, it was designated as *Rj*_2_, the second *Rj* genotype to be identified. Several novel *Rj* genotypes controlled by the dominant allele were subsequently reported, exhibiting similar traits to *Rj*_2_, including the *Rj*_3_ genotype, a nodulation-restriction trait associated with soybean Hardee and *Bradyrhizobium elkanii* USDA33 (Vest, 1970), followed by *Rj*_4_, a nodulation-restriction trait associated with soybean Hill and *B. elkanii* USDA61 (Vest and Caldwell, 1972), and *Rfg1*, a nodulation-restriction trait with soybean Williams 82 and certain strains of the *Ensifer* genus (Devine and Kuykendall, 1994). Zhang *et al.* (2021) described *GmNNL1*, a nodulation-restriction trait with soybean Heng Feng Wu Dou and certain strains of the *Bradyrhizobium* genus, particularly root hair infection caused by effector-triggered immunity (ETI).

Other recessive alleles, specifically *rj*_5_/*rj*_6_, exhibit nodulation-deficient phenotypes analogous to that of *rj*_1_; however, both genetic loci (*rj*_5_ and *rj*_6_) are responsible for this recessive phenotype (Pracht *et al.*, 1993). Interestingly, *rj*_7_, for example, the soybean lines NOD1-3 and En6500, displays a hypernodulation trait and is also a recessive allele (Vuong *et al.*, 1996). Although Vuong *et al.* (1996) designated soybean line NOD2-4 as *rj*_8_, Vuong and Harper (2000) later revealed that this *rj*_8_ genotype was controlled by the *rj*_7_ locus.

## Mechanisms for the enhancement and restriction of nodule formation in *Rj* genotypes

### *rj*_1_, *rj*_5_/*rj*_6_: nodulation-deficient traits with various rhizobial strains

Authentication with rhizobial Nod factors (NFs) and soybean Nod factor receptors (NFRs) is essential for nodule‍ ‍formation in soybean, as well as in the genera *Bradyrhizobium* and *Ensifer* (Devine *et al.*, 1980; Francisco and Akao, 1993; Rasooly and Isleib, 1993; Radutoiu *et al.*, 2003). NFR1 and NFR5 form the NFR complex, which is considered to be necessary for the reception and recognition of NF.

In the *rj*_1_ and *rj*_5_/*rj*_6_ genotypes, most rhizobia fail to induce nodule formation due to a loss of function in the NFR complex. The soybean cultivar Bragg contains the *NFR1α* and *NFR1β* genes; however, the expression level of *NFR1β* is significantly lower than that of *NFR1α*, with 70% being splice variants of *NFR1β*. Therefore, the *NFR1α* mutant (*rj*_1_) exhibits a nodulation-deficient trait despite the presence of *NFR1β* (Indrasumunar *et al.*, 2011).

*NFR1α* encodes a lysine motif (LysM) receptor-like kinase that localizes in the plasma membrane of plant cells. It features an extracellular LysM receptor domain, transmembrane domain, and intracellular Ser/Thr kinase domain (Madsen *et al.*, 2011). This kinase domain exhibits phosphorylation activity (Madsen *et al.*, 2011). Clark-*rj*_1_, a *rj*_1_ soybean experimental line, has a frameshift mutation near the transmembrane domain (Indrasumunar *et al.*, 2011). En1282, another *rj*_1_ soybean, also harbors a mutation in *NFR1*α (Ikeda *et al.*, 2010).

*NFR5α*/*NFR5β*, which encode receptor-like kinase (NFR5), are responsible for the *rj*_5_/*rj*_6_ traits. Unlike NFR1, NFR5 does not exhibit phosphorylation activity (Arrighi *et al.*, 2006; Madsen *et al.*, 2011). The NFR5α and NFR5β proteins are both functional in the perception and recognition of NF (Indrasumunar *et al.*, 2010). A nodulation-deficient trait is only observed when mutations occur in both‍ ‍the *NFR5α* and *NFR5β* genes (Pracht *et al.*, 1993; Indrasumunar *et al.*, 2010).

Soybean has a large genome size (1.1 Gb) and is a paleopolyploid with approximately 75% of its genes maintaining multiple copies (Schmutz *et al.*, 2010). Due to this complexity, the identification of the *rj*_1_ locus and *rj*_5_/*rj*_6_ locus was strongly facilitated by mutations in and the isolation of genes of *Lotus japonicus*, a model legume (Schauser *et al.*, 1998; Radutoiu *et al.*, 2003). Comparative ana­lyses with these pioneering studies made significant contributions to‍ ‍our understanding of soybean nodulation genetics (Indrasumunar *et al.*, 2010; Indrasumunar *et al.*, 2011).

## Rhizobial strains overcoming *rj*_1_

**Fig. 1.**
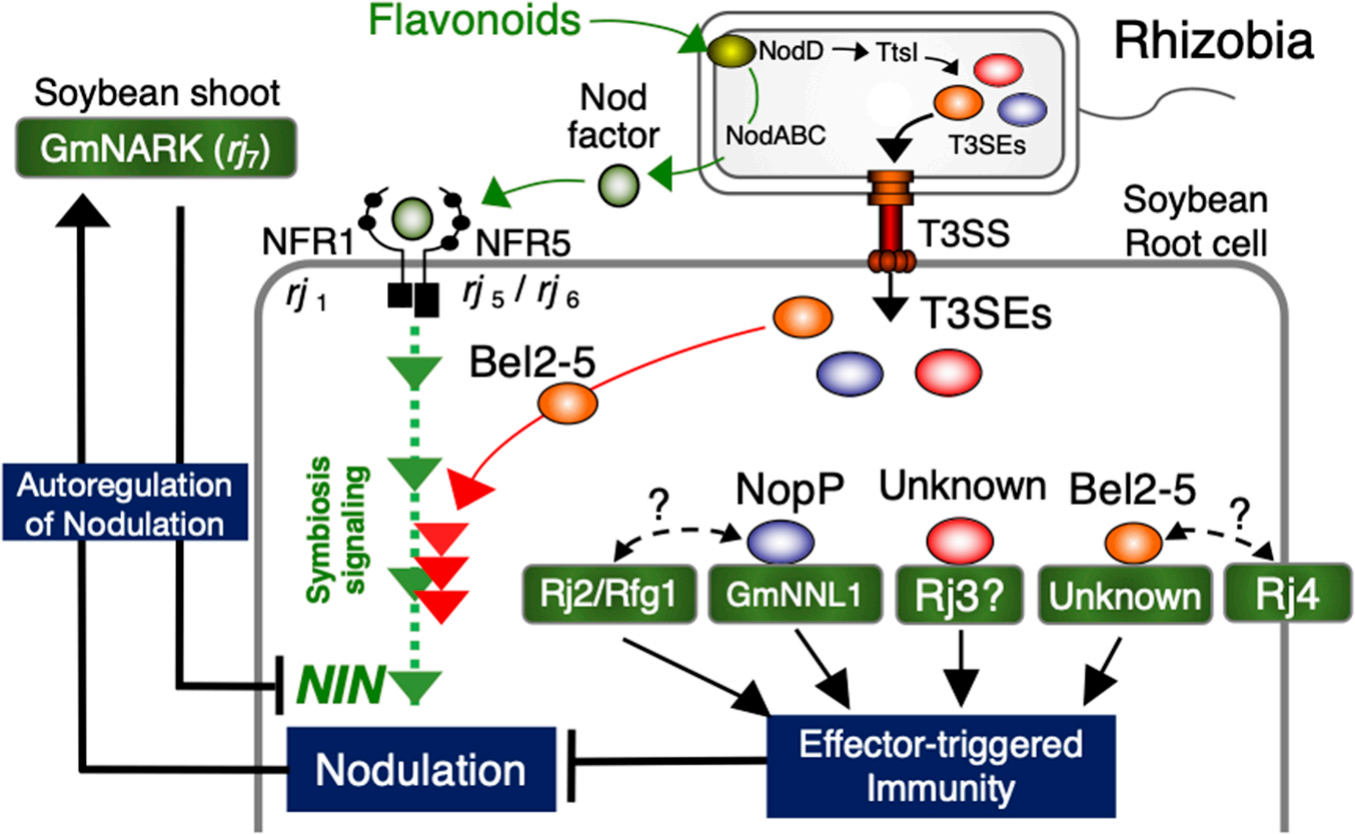
Function of soybean *Rj* genes and rhizobial Type III secreted effectors in nodulation and defense signaling. Flavonoids derived from host legumes induce the production of Nod factors in rhizobia. Upon the recognition of these Nod factors (NFs) by Nod factor receptors (NFRs), a symbiotic signaling pathway is activated, leading to nodule formation. One of the Type III secreted effectors (T3SEs) Bel2-5 from rhizobia enhances nodule formation by bypassing the recognition of NFs and directly activating the NF signaling pathway. The number of nodules per plant is regulated by a mechanism known as autoregulation of nodulation, which involves GmNARK. Conversely, some T3SEs, such as NopP and Bel2-5, are recognized by the host factors, leading to the induction of effector-triggered immunity, which ultimately blocks nodulation or inhibits nodulation.

Most rhizobial strains are unable to induce nodule formation in *rj*_1_ soybeans; however, previous studies reported the nodulation ability of specific rhizobia, such as *B. elkanii* USDA61, in these soybeans (Murphy and Elkan, 1965; Devine and Weber, 1977) (Table 1). Although the mechanisms underlying nodulation in *rj*_1_ soybeans remain unclear, the Type III Secretion System (T3SS) of USDA61 has been identified as a crucial rhizobial factor (Okazaki *et al.*, 2013). T3SS is a protein secretion system found in a wide range of Gram-negative bacteria, which secretes various proteins known as effectors (Type III Secreted Effectors; T3SEs) into the extracellular space or directly into host cells (Hueck, 1998). T3SEs are recognized as virulence factors in plant and animal pathogens, and also contribute to rhizobial infection and nodule maturation in nodule symbiosis (Büttner and Bonas, 2003; Deakin and Broughton, 2009). Bel2-5, one of the T3SEs in USDA61, has been identified as the effector responsible for inducing nodulation symbiosis with *rj*_1_ soybeans (Okazaki *et al.*, 2013; Ratu *et al.*, 2021b) (Table 1). Bel2-5 was originally identified as a rhizobial factor that induces symbiotic incompatibility with *Rj*_4_ soybeans (Faruque *et al.*, 2015) (Fig. 1). This finding is significant in the context of the co-evolution of soybean and rhizobia.

Only intercellular infection has been observed in the nodulation of *rj*_1_ soybeans (Pueppke and Payne, 1987; Okazaki *et al.*, 2013). Intercellular infection is regarded as a more primitive form of infection (Madsen *et al.*, 2010). These findings suggest that T3SS-mediated nodulation in rhizobia represents a primitive nodulation mechanism that precedes the NF-dependent process; however, definitive evidence currently remains limited (Ibáñez *et al.*, 2017; Ratu *et al.*, 2023). Additionally, the precise mechanisms, including the specific signaling pathways induced within host legume cells and the host plant factors involved, have yet to be elucidated.

## *Rj*_2_/*Rfg1*, *Rj*_3_, and *Rj*_4_: nodulation-restriction traits associated with specific rhizobial strains

The *Rj*_2_/*Rfg1*, *Rj*_3_, and *Rj*_4_ genotypes exhibit nodulation-restriction traits in response to rhizobial strains that possess specific T3SEs. *Rj*_2_/*Rfg1* and *Rj*_4_ are considered to induce ETI, a robust plant defense response (Yasuda *et al.*, 2016; Shine *et al.*, 2019). Recent studies have investigated the interactions between soybean genes and rhizobial T3SEs (Table 1, Fig. 1).

## NopP and its role in symbiotic incompatibilities with *Rj*_2_/*Rfg1* genotypes

*Rj*_2_ soybeans exhibit a nodulation-restriction trait with specific strains of *Bradyrhizobium*, while the *Rfg1* genotype shows a similar trait with certain strains of *Ensifer* (Yang *et al.*, 2010) (Table 1). *Rj*_2_ was initially identified in the soybean cultivar Hardee, which associates with *B. diazoefficiens* USDA122, and is characterized by the formation of not only a limited number of nodules, but also numerous bump-like structures on the roots (Caldwell, 1966). The *Rfg1* genotype is incompatible with *Ensifer fredii* USDA205 (Devine and Kuykendall, 1994).

The *Rj*_2_/*Rfg1* genotypes exhibit incompatibility with rhizobia possessing the effector NopP (Sugawara *et al.*, 2018; Rehman *et al.*, 2019) (Table 1). Specifically, *Rj*_2_ soybeans blocked nodulation by bradyrhizobial strains containing the critical amino acid combination of R60, R67, and H173. Conversely, *Rfg1* soybeans inhibited species of the *Ensifer* genus that also contain NopP; however, the critical characteristics remain unclear.

The genes responsible for *Rj*_2_ and *Rfg1* encode resistance protein (R protein) possessing a TIR-NBS-LRR domain, which is characteristic of disease response mechanisms (Yang *et al.*, 2010). While the *Rj*_2_ and *Rfg1* genotypes both‍ ‍cause incompatibility with specific strains of *Bradyrhizobium* and *Ensifer*, they share the same genetic locus. The critical amino acid combination required for incompatibilities differ between the two genotypes (Fan *et al.*, 2017; Sugawara *et al.*, 2019). In the Rj2 protein, the amino acid combination of E452 and I490 is essential for *Rj*_2_-mediated incompatibility. In contrast, the Rfg1 protein requires E731, N736, S743, D756, and S758 for *Rfg1*-mediated incompatibility. These findings have led to the successful construction of functionally expanded Rj2/Rfg1 proteins (Fan *et al.*, 2017; Sugawara *et al.*, 2019; Li *et al.*, 2023), and suggest that a more detailed understanding of the mole­cular mechanisms underlying *Rj* genotypes will facilitate the artificial manipulation of nodulation symbiosis.

## The *GmNNL1* genotype is triggered by NopP, but shows different phenotypes with *Rj*_2_/*Rfg1*

While *GmNNL1* shares NopP as a rhizobial factor with *Rj*_2_/*Rfg1*, GmNNL1 directly recognizes NopP and the characteristics of symbiotic incompatibility differ from those of *Rj*_2_/*Rfg1* (Zhang *et al.*, 2021) (Table 1). GmNNL1 is an R protein primarily expressed in soybean root hairs, and its symbiotic incompatibility is characterized by the inhibition of rhizobial infection from root hairs (inhibition of infection thread formation from root hairs), resulting in the formation of some nodules by crack entry (Zhang *et al.*, 2021).

## Relationships between rhizobial T3SS and *Rj*_3_

*Rj*_3_, one of the *Rj* genotypes identified in some soybean cultivars, such as Hardee and D-51, is characterized by the ability of specific rhizobial strains to induce only a limited number of nodules (Vest, 1970). Although the factors responsible in both soybean and rhizobia remain unknown, Shobudani *et al.* (2020) reported the involvement of rhizobial T3SS in this symbiotic incompatibility, similar to the mechanisms observed in *Rj*_2_/*Rfg1* and *Rj*_4_ (Table 1).

## Bel2-5 and its role in symbiotic incompatibility with the *Rj*_4_ genotype

*Rj*_4_, which is found in soybean cultivars such as Hill and Fukuyutaka, strongly suppresses nodulation with specific *Bradyrhizobium* strains, such as USDA61 (Hayashi *et al.*, 2014; Tang *et al.*, 2016). The plant factor associated with this incompatibility is the *Rj*_4_ gene, which encodes a thaumatin-like protein, while the rhizobial factor is identified as the effector Bel2-5 (Faruque *et al.*, 2015; Tsurumaru *et al.*, 2015) (Table 1). Bel2-5 may induce nodulation in *rj*_1_ soybeans. Ratu *et al.* (2021a) reported that nearly all domains of Bel2-5 were crucial for both nodulation in *rj*_1_ and symbiotic incompatibility in *Rj*_4_. These findings suggest that Bel2-5 plays an important role in host soybean cells; however, further studies are warranted to obtain more information and elucidate the underlying mechanisms.

## Hypernodulation in *rj*_7_ and autoregulation of nodulation (AON)

The hypernodulation mutant line *rj*_7_, also known as nitrate-tolerant symbiotic (NTS), is characterized by well-nodulated plants under high nitrate conditions, which ordinarily suppress nodulation (Carroll *et al.*, 1985a, 1985b; Clark *et al.*, 1997). The mechanisms underlying nodule formation associated with *rj*_7_ have been elucidated primarily through research utilizing the model leguminous plant *L. japonicus* (Kawaguchi, 2016; Okuma and Kawaguchi, 2021). Furthermore, the mechanisms responsible for the suppression of nodulation under high nitrate conditions have been clarified using *L. japonicus* (Misawa *et al.*, 2022).

The *rj*_7_ genotype, a recessive trait, is associated with the long-distance control of nodulation through shoot-root communication, referred to as AON (Clark *et al.*, 1997; Kawaguchi, 2016). This control mechanism is similar to the CLAVATA (CLV) signaling pathway, which regulates plant stem cell maintenance in *Arabidopsis thaliana* (Searle *et al.*, 2003; Oka-Kira and Kawaguchi, 2006; Suzaki and Nishida, 2016). The corresponding gene for *rj*_7_ is the nodule autoregulation receptor kinase (NARK) gene, which encodes an LRR-type receptor kinase homologous to HYPERNODULATION ABERRANT ROOT FORMATION 1 (HAR1) in *L. japonicus* (Nishimura *et al.*, 2002) (Table 1).

HAR1, a receptor kinase discovered in *L. japonicus*, functions in the leaf phloem and recognizes signal peptides from the root (Krusell *et al.*, 2002; Nishimura *et al.*, 2002; Okamoto *et al.*, 2009). In *rj*_7_ soybeans, mutations in the *NARK* gene have been linked to the loss of AON and excessive nodulation (Nishimura *et al.*, 2002; Schnabel *et al.*, 2005; Lin *et al.*, 2010).

Numerous studies have reported relationships between the presence of nitrate and the control of rhizobial infection and nodulation. Therefore, nitrate-tolerant lines, such as the *rj*_7_ genotype, may serve as important research resources for enhancing the coexistence and functionality of nitrogen fertilizers and rhizobial nodulation symbiosis in legume crops.

## Perspectives for agricultural applications and concluding remarks

### The *Rj* genotype utilization strategy for reducing greenhouse gas emissions from soybean cultivation

**Fig. 2.**
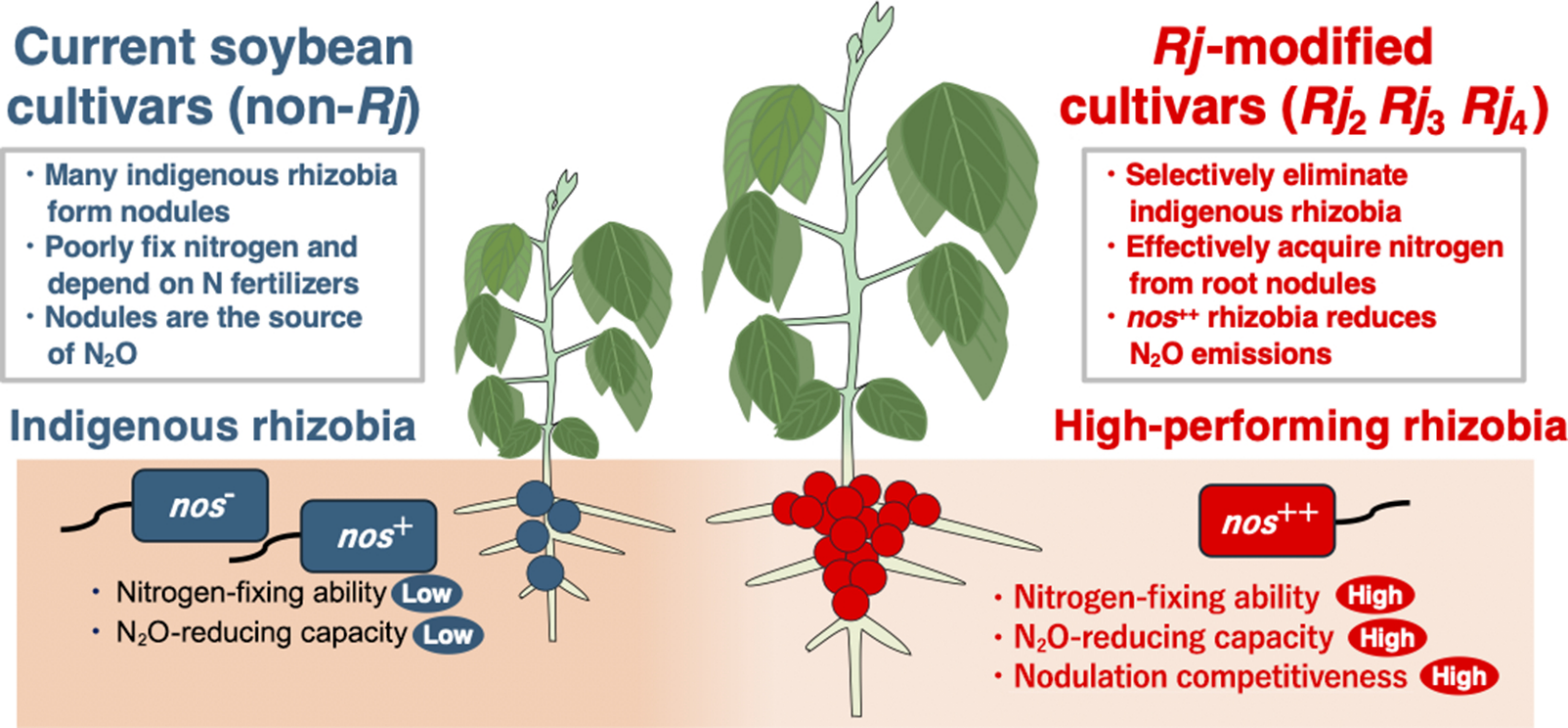
Agricultural utilization of *Rj* soybeans and high-performing rhizobia. Indigenous rhizobia in soils generally exhibit low nitrogen-fixing capabilities, and soybean varieties lacking *Rj* genes are often dominated by these indigenous rhizobia. This leads to reduced nitrogen-fixing efficiency, increased reliance on nitrogen fertilizers, and contributes to elevated emissions of N_2_O. One strategy to mitigate this issue is the utilization of *Rj*-modified soybeans in conjunction with superior rhizobia. Soybean experimental lines that accumulate multiple *Rj* genotypes may effectively exclude indigenous rhizobia, thereby allowing for preferential colonization by high-performing rhizobia that possess a high N_2_-fixing ability and N_2_O-reducing capacity. The cultivation of these *Rj*-modified soybeans with efficient *nos*^++^ rhizobia in the field is expected to enhance nitrogen fixation efficiency, reduce fertilizer application, and decrease N_2_O emissions.

Nitrogen-containing chemical fertilizers have been fundamental to food production, supporting explosive population growth and helping to avert the food crisis feared since the late 19th century (Crookes, 1898; Erisman *et al.*, 2008). However, the large-scale application of nitrogen fertilizers results in a denitrification process mediated by microorganisms, ultimately releasing nitrogen gas (N_2_) and nitrous oxide (N_2_O) into the atmosphere. N_2_O has a significant global warming potential, approximately 300-fold greater than carbon dioxide. Tian *et al.* (2024) estimated that N_2_O released directly and indirectly from agricultural activities constituted approximately 74% of anthropogenic emissions in 2010s. Therefore, measures to reduce greenhouse gas emissions in the agricultural sector are imperative (Yoro and Daramola, 2020; Hassan *et al.*, 2022). Field studies have shown that soils under leguminous crops, particularly soybeans, exhibit high N_2_O emissions derived from plant residues (Yang and Cai, 2005). One effective strategy for mitigating N_2_O emissions from soybean fields is the utilization of *Rj* soybean cultivars in conjunction with N_2_O-reducing rhizobia. Some rhizobia possess nitrous oxide reductase (Nos), which facilitates the conversion of N_2_O to nitrogen gas (N_2_). Among these, *Bradyrhizobium ottawaense* exhibited high N_2_O-reducing activity (Wasai-Hara *et al.*, 2023). However, indigenous rhizobia are typically abundant in soil, competing with inoculated strains and potentially hindering their colonization. This challenge may be addressed through the combined use of rhizobial materials with high N_2_O-reducing activity (*nos*^++^ strains) and *Rj*-modified soybean cultivars, which selectively associate with these rhizobial materials (Fig. 2).

Shiro and Saeki (2022) conducted field trials using soybean experimental lines that accumulated *Rj* genotypes (*Rj*_2_/*Rj*_3_/*Rj*_4_). They also performed inoculations with functionally superior rhizobia, specifically *B. diazoefficiens* USDA110. The findings obtained suggested that the accumulation of *Rj*‍ ‍genotypes along with effective rhizobial inoculants enhanced the occupancy rate of functionally superior rhizobia and improved soybean productivity. Therefore, *Rj* genotypes have potential for the artificial control of nodulation symbiosis. Furthermore, strategies that integrate effective rhizobial inoculants with *Rj* soybeans may contribute to reductions in N_2_O emissions from agricultural fields globally.

## Application of nodulation capacity to non-leguminous crops

Nitrogen supply to non-leguminous crops, such as rice, is now primarily achieved through the application of nitrogen fertilizers and soil nitrogen enhancements via green manure (Erisman *et al.*, 2008; Kebede, 2021). Some bradyrhizobia are recognized as endophytic bacteria that promote rice growth (Chaintreuil *et al.*, 2000; Greetatorn *et al.*, 2019). These findings suggest the potential of applying the nodulation abilities of rhizobia to non-leguminous crops through the T3SS/Bel2-5 mechanism. This concept involves the direct application of the nodulation ability mediated by Bel2-5 from *rj*_1_ soybeans to non-leguminous crops. While significant challenges remain in the practical application of this unique mechanism, elucidating the mole­cular interactions between Bel2-5 and soybean factors is promising for the development of technologies that confer nodulation capabilities to non-leguminous crops, such as rice.

We herein investigated the interplay between *Rj* soybeans and rhizobia. Recent research revealed the mole­cular mechanisms governing the interaction between *Rj* soybeans and rhizobial T3SEs, as well as the connections between *Rj* genotypes and plant immunity systems. Future studies need to focus not only on the identification and classification of novel *Rj* genotypes, but also on elucidating the mole­cular mechanisms that regulate symbiotic enhancement and suppression. These fundamental insights are expected to facilitate the development of innovative technologies that enhance soybean productivity while minimizing environmental impacts.

## Citation

Fukunaga, S., Ratu, S. T. N., and Okazaki, S. (2025) Regulation of Root Nodule Symbiosis by Soybean *Rj* Genotypes and Rhizobial Effectors. *Microbes Environ ***40**: ME25027.

https://doi.org/10.1264/jsme2.ME25027

## Figures and Tables

**Table 1.** *Rj* genotypes and responsible genes in soybean cultivars/mutant lines.

Soybean cultivars, mutant lines	*Rj* genotype	Soybean genes	Feature of soybean genes	Rhizobial strains	Rhizobial factors	References
**Non-nodulation**						
Clark-*rj*_1_, En1282	*rj*_1_	*NFR1α*	Receptor-like kinase	All (except some strains)	Bel2-5 (T3SS effector)	Indrasumunar *et al.*, 2011; Ratu *et al.*, 2021b
NN5, nod139	*rj*_5_/*rj*_6_	*NFR5α*/*NFR5β*	Receptor-like kinase	—	—	Indrasumunar *et al.*, 2010; Pracht *et al.*, 1993
**Suppress nodulation with specific rhizobial species**						
Hardee, CNS	*Rj*_2_	*Rj2*	TIR-NBS-LRR	USDA122 (*B. diazoefficiens*)	NopP (T3SS effector)	Yang *et al.*, 2010; Sugawara *et al.*, 2019
Williams 82	*Rfg1*	*Rfg1*	TIR-NBS-LRR	USDA205 (*Ensifer fredii*)	NopP (T3SS effector)	Devine and Kuykendall, 1994; Yang *et al.*, 2010; Rehman *et al.*, 2019
Hardee, D-51	*Rj*_3_	Unknown	—	USDA33 (*B. elkanii*), BLY3-8 (*B. elkanii*)	Unknown (T3SS effector?)	Vest, 1970; Shobudani *et al.*, 2020
Hill, Fukuyutaka	*Rj*_4_	*Rj4*	Thaumatin-like protein	USDA61 (*B. elkanii*), Is-34 (*B. japonicum*)	Bel2-5 (T3SS effector)	Hayashi *et al.*, 2014; Faruque *et al.*, 2015; Tang *et al.*, 2016
Heng Feng Wu Dou	*GmNNL1*	*GmNNL1*	TIR-NBS-LRR	USDA110 (*B. diazoefficiens*), USDA6 (*B. japonicum*)	NopP (T3SS effector)	Zhang *et al.*, 2021
**Hypernodulation**						
NOD1-3, En6500, (NOD2-4)	*rj*_7_ (*rj*_8_)	*GmNARK*	Receptor-like kinase	—	—	Vuong *et al.*, 1996; Vuong and Harper, 2000; Searle *et al.*, 2003;
